# Merozoite surface protein-1 genetic diversity in *Plasmodium malariae* and *Plasmodium brasilianum* from Brazil

**DOI:** 10.1186/s12879-015-1238-8

**Published:** 2015-11-16

**Authors:** Lilian O. Guimarães, Gerhard Wunderlich, João M. P. Alves, Marina G. Bueno, Fabio Röhe, José L. Catão-Dias, Amanda Neves, Rosely S. Malafronte, Izilda Curado, Wilson Domingues, Karin Kirchgatter

**Affiliations:** Núcleo de Estudos em Malária, Superintendência de Controle de Endemias/Instituto de Medicina Tropical, Universidade de São Paulo, São Paulo, SP 05403-000 Brazil; Departamento de Parasitologia, Instituto de Ciências Biomédicas, Universidade de São Paulo, São Paulo, SP 05508-900 Brazil; Departamento de Patologia, Faculdade de Medicina Veterinária e Zootecnia, Universidade de São Paulo, São Paulo, SP 05508-270 Brazil; Wildlife Conservation Society, Rio de Janeiro, RJ 22461-000 Brazil; Laboratório de Protozoologia, Instituto de Medicina Tropical, Universidade de São Paulo, São Paulo, SP 05403-000 Brazil; Departamento de Moléstias Infecciosas e Parasitárias, Faculdade de Medicina, Universidade de São Paulo, São Paulo, SP 01246-903 Brazil; Laboratório de Imunoepidemiologia, Superintendência de Controle de Endemias, São Paulo, SP 01027-000 Brazil; Laboratório de Soroepidemiologia e Imunobiologia, Instituto de Medicina Tropical de São Paulo, Universidade de São Paulo, São Paulo, SP 05403-000 Brazil

**Keywords:** Malaria, *Plasmodium malariae*, Genetic diversity, Merozoite surface protein 1, Brazil, Atlantic forest

## Abstract

**Background:**

The merozoite surface protein 1 (MSP1) gene encodes the major surface antigen of invasive forms of the *Plasmodium* erythrocytic stages and is considered a candidate vaccine antigen against malaria. Due to its polymorphisms, MSP1 is also useful for strain discrimination and consists of a good genetic marker. Sequence diversity in MSP1 has been analyzed in field isolates of three human parasites: *P. falciparum*, *P. vivax*, and *P. ovale*. However, the extent of variation in another human parasite, *P. malariae*, remains unknown. This parasite shows widespread, uneven distribution in tropical and subtropical regions throughout South America, Asia, and Africa. Interestingly, it is genetically indistinguishable from *P. brasilianum*, a parasite known to infect New World monkeys in Central and South America.

**Methods:**

Specific fragments (1 to 5) covering 60 % of the MSP1 gene (mainly the putatively polymorphic regions), were amplified by PCR in isolates of *P. malariae* and *P. brasilianum* from different geographic origin and hosts. Sequencing of the PCR-amplified products or cloned PCR fragments was performed and the sequences were used to construct a phylogenetic tree by the maximum likelihood method. Data were computed to give insights into the evolutionary and phylogenetic relationships of these parasites.

**Results:**

Except for fragment 4, sequences from all other fragments consisted of unpublished sequences. The most polymorphic gene region was fragment 2, and in samples where this region lacks polymorphism, all other regions are also identical. The low variability of the *P. malariae msp1* sequences of these isolates and the identification of the same haplotype in those collected many years apart at different locations is compatible with a low transmission rate. We also found greater diversity among *P. brasilianum* isolates compared with *P. malariae* ones. Lastly, the sequences were segregated according to their geographic origins and hosts, showing a strong genetic and geographic structure.

**Conclusions:**

Our data show that there is a low level of sequence diversity and a possible absence of allelic dimorphism of MSP1 in these parasites as opposed to other *Plasmodium* species. *P. brasilianum* strains apparently show greater divergence in comparison to *P. malariae*, thus *P. malariae* could derive from *P. brasilianum*, as it has been proposed.

**Electronic supplementary material:**

The online version of this article (doi:10.1186/s12879-015-1238-8) contains supplementary material, which is available to authorized users.

## Background

Malaria is a mosquito-borne disease that, in humans, is caused by five different species of *Plasmodium.* Most cases are caused by either *P. falciparum* or *P. vivax*, but human infections can also be caused by *P. ovale*, *P. malariae*, and, in parts of Southeast Asia, by *P. knowlesi*, a monkey malaria [1]. *P. malariae* is also very closely related to a malarial parasite that infects 12 genera of New World primates, *P. brasilianum* [2]. *P. malariae* appears to be genetically indistinguishable from *P. brasilianum*, although complete genome data supporting this conclusion is still unavailable. This points to a recent host transfer between humans and monkeys, but the direction of transfer is still controversial [3–5].

*P. malariae* is a cosmopolitan parasite which develops where the summer isotherm does not fall below 15 °C (59 °F). Its distribution is variable and spotty [2]. It has been identified throughout tropical regions in Africa, Asia, and South America. *P. brasilianum* has been reported in South and Central America. There are reports of natural infections of monkeys in Panama, Venezuela, Colombia, Peru, Brazil, and French Guiana [2, 6]. In Brazil, *P. malariae* and *P. brasilianum* are sparsely distributed in the Amazon Region and also in extra-Amazonian Atlantic forest regions, where malaria transmission is considered hypoendemic and controlled [7–9]. This tropical rainforest has an ample diversity of bromeliads that are an ideal environment for *Anopheles* mosquitoes of the subgenus *Kerteszia*, mainly *An. (K.) cruzii*, which use the axils of these plants as larval habitat [10]. In this region, although only a small number of clinical cases of malaria are registered, a large portion of the population shows serological evidence of recent exposure to variants of *P. vivax* or *P. malariae* [11], suggesting a high prevalence of asymptomatic infection. These asymptomatic cases are often missed or underestimated because control programs are focused on passive and active investigation of symptomatic cases.

The merozoite surface protein 1 (MSP1) is the major surface antigen of invasive forms of the erythrocytic stages of *Plasmodium* and has been proposed as a vaccine antigen against malaria [12]. However, an extensive polymorphism in this protein was found in both *P. falciparum* and *P. vivax* from different geographical areas, representing a major obstacle to the development of an effective vaccine [13, 14]. Although the sequences from *P. falciparum* and *P. vivax* MSP1 genes have been described a long time ago [15, 16], *msp1* genes were more recently identified in *P. malariae* and *P. ovale* [17]. Recently, a study showed low levels of sequence diversity in MSP1 of *P. ovale* among Thai isolates [18]. However, until now, only two studies of *P. malariae* and/or *P. brasilianum msp1* sequences were performed. The first one, using only ~200 bp in the N-terminal region of the gene, showed that *P. brasilianum* has limited polymorphism in French Guiana [6]. A low variability (97 to 100 % identity) was also found using a longer fragment (~600 bp), also in the N-terminal region, between seven *P. brasilianum* sequences from monkeys of the state of Rondônia (Western Brazilian Amazon) [19].

MSP1 genes from *Plasmodium* species infecting mammals exhibit sizes of ~5 kb. The single copy *msp1* gene of *P. falciparum* and *P. vivax* is divided into blocks, based on analysis of interspecies sequence diversity: seven highly variable blocks are interspersed with ten conserved regions [20]. Although there is no evidence that the *msp1* sequences from all species could be divided in blocks, regions of interspecies conservation and variability across the entire MSP1 protein can be clearly identified [17]. In this study, the genetic polymorphism of five *msp1* gene regions, adding up to ~3000 bp, was analyzed among field isolates collected from different hosts in Brazil. These data were used to examine genetic diversity in relation to geographic origin and hosts.

## Methods

### Samples

Seventeen samples of *P. malariae/P. brasilianum* genomic DNA were used in this study (Table 1). Regarding the human samples, three were obtained in the Amazon Region (samples 23PA, 50PA, and 66PA) and eight were collected in the Atlantic forest (A, I11, 58, 72, 23a, 190, 157a, and 222a). Among these last samples, two were obtained from patients involved in a case of transfusion malaria: one sample was obtained from a splenectomized patient (58) who was infected by blood transfusion and another from her blood donor, an asymptomatic carrier (72), who probably acquired the infection in the location of Palestina, Juquitiba [21]. The remaining four samples (23a, 190, 157a, and 222a) were obtained during malaria control activities of this malaria focus. The non-human samples analyzed were: one *P. malariae/P. brasilianum* sample obtained from a pool of three *Anopheles Kerteszia cruzii* mosquitoes collected in Itanhaém (Atlantic forest region) and four *P. brasilianum* samples obtained from simian hosts captured in Igapó-Açú, roughly 300 km from Manaus (AM) (*Callicebus caligatus*) and Abunã, roughly 200 km from Porto Velho (RO) (*Callicebus dubius* and *Pithecia sp*.), both located in the Brazilian Amazon. The Peruvian III strain of *P. brasilianum* isolated from *Saimiri sciureus* monkeys of Iquitos in 1987 was used as a control [22]. For all the samples collected, the presence of *P. malariae/P. brasilianum* was verified using two PCR protocols and sequencing: (i) a nested PCR amplifying a small subunit ribosomal RNA (ssrRNA) based on the technique originally described by Snounou et al. [23, 24], in which the first amplification reaction was modified in order to increase its sensitivity [25]; and (ii) a nested PCR amplifying the mitochondrial cytochrome b gene (*cytb*) [26].

**Table 1:** Human and non-human samples used in this study

Human sample	Year of Collection	Origin	
23PA	1996	Peixoto de Azevedo, MT, Amazon Region, BR	
50PA	1996	Peixoto de Azevedo, MT, Amazon Region, BR	
66PA	1996	Peixoto de Azevedo, MT, Amazon Region, BR	
A	2000	Iguape, SP, Atlantic Forest, BR	
I11	2002	Iporanga, SP, Atlantic Forest, BR	
58	2004	São Paulo, SP (Blood transfusion from Patient 72)	
72	2004	Juquitiba, SP, Atlantic Forest, BR	
23a	2005	Juquitiba, SP, Atlantic Forest, BR	
190	2006	Juquitiba, SP, Atlantic Forest, BR	
157a	2007	Juquitiba, SP, Atlantic Forest, BR	
222a	2007	Juquitiba, SP, Atlantic Forest, BR	
Sample	Year of Collection	Source	Origin
PIII	1987	*Saimiri sciureus*	Iquitos, Peru
M95	2010	*Anopheles (K.) cruzii*	Itanhaém, SP, Atlantic Forest, BR
P169	2010	*Callicebus dubius*	Porto Velho, RO, Amazon Region, BR
P171	2010	*Pithecia sp.*	Porto Velho, RO, Amazon Region, BR
P177	2011	*Callicebus caligatus*	Manaus, AM, Amazon Region, BR
P182	2011	*Callicebus caligatus*	Manaus, AM, Amazon Region, BR

*SP* São Paulo state, *AC* Acre state, *RO* Rondônia state, *AM* Amazonas state, *BR* Brazil

### Design of PmMSP1-specific primers

**Fig. 1:**
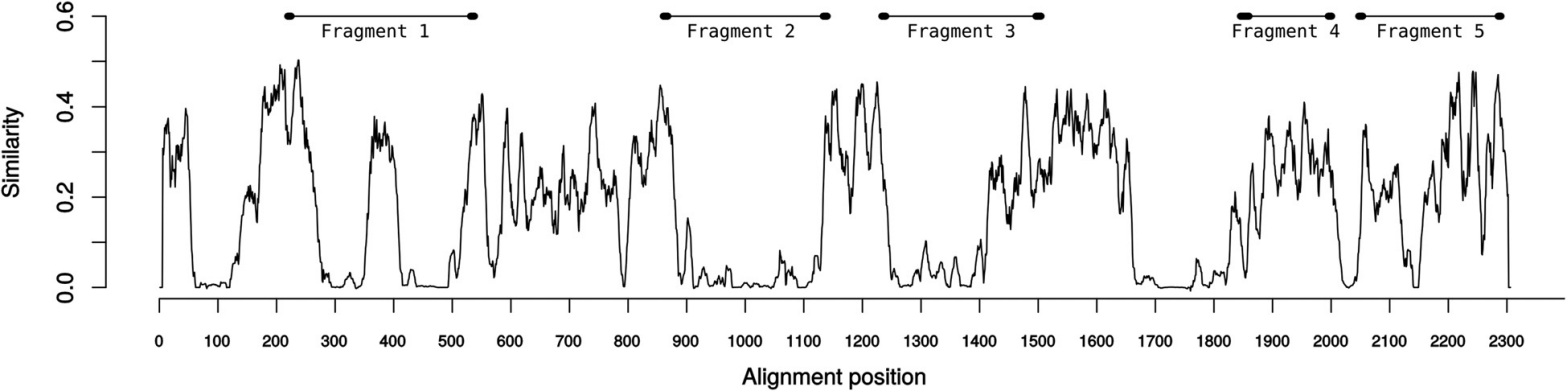
Interspecies comparison of MSP1 protein sequences. Full-length deduced MSP1 amino acid sequences from 16 *Plasmodium* species (Table 2) were aligned and an average amino acid similarity score was determined by plotcon as described in Methods. Each sequence fragment studied in this work is indicated above the graph, and location and length of PCR oligonucleotides are indicated by the ovals at the ends of fragments

Ideally, primer design would have been guided by an alignment of *P. malariae*/*P. brasilianum msp1* sequences; however, only one complete *msp1* sequence has been published. Therefore, we have used an alignment of *msp1* from different *Plasmodium* species. We assume that regions conserved among different species are possibly also conserved within the same species. Thus, to focus our efforts on parts most likely to be genetically diverse, we performed an alignment for determination of percent amino acid sequence identity among nineteen MSP1 sequences, representing 16 *Plasmodium* species, collected from GenBank (Table 2). Calculation of amino acid sequence similarity levels was done using the plotcon program from the EMBOSS package [27], with plotting performed in R. Three regions with the lowest similarities among *Plasmodium* species were chosen. Additionally, two regions with high similarities interspecies were also chosen including the C-terminal region of the MSP1 (MSP1_19_), the leading vaccine candidate. Therefore, five regions of the *msp1* gene were chosen for our analysis (Fig. 1).

**Table 2:** Merozoite surface protein 1 (MSP1) sequences of *Plasmodium* species

		GenBank accession no.	
*Plasmodium* species (strain)	Host species	Aminoacid	Nucleotide
*berghei*	*Grammomys surdaster*	AAF13063.1	AF187232.1
*chabaudi*	*Thamnonys rutilans*	AAA29499.1	L22982.1
*coatneyi*	*Macaca fascicularis*	BAF74048.1	AB266180.1
*cynomolgi*	*Macaca sinica*	BAF74063.1	AB266195.1
*falciparum* (MAD20)	*Homo sapiens*	A26868	X05624.2
*falciparum* (K1)	*Homo sapiens*	CAA27070.1	X03371.1
*fragile*	*Macaca sinica*	BAF74049.1	AB266181.1
*gallinaceum*	*Gallus gallus*	CAH10838.1	AJ809338.1
*hylobati*	*Hylobati moloch*	BAF74050.1	AB266182.1
*inui*	*Cynopithecus niger*	BAF74051.1	AB266183.1
*knowlesi*	*Macaca fascicularis*	BAF74052.1	AB266184.1
*malariae* (MM1A)	*Homo sapiens*	ACZ51237.1	FJ824669
*ovale* (OM1A)	*Homo sapiens*	ACZ51238.1	FJ824670
*ovale* (OM1B)	*Homo sapiens*	ACZ51239.1	FJ824671
*reichenowi*	*Pan troglodytes*	CAH10285.1	AJ786604.1
*simiovale*	*Macaca sinica*	BAF74053.1	AB266185.1
*vivax* (Belem)	*Homo sapiens*	AAN86208.1	AF435594.1
*vivax* (Sal1)	*Homo sapiens*	EDL45115.1	AAKM01000007.1
*yoelii*	*Thamnonys rutilans*	EAA17822.1	AABL01001865.1

To obtain *P. malariae*/*P. brasilianum*-specific primers from the MSP1 gene, nucleotide sequences flanking the five regions were designed based on the only sequence available for *P. malariae* (MM1A isolate from Cameroon, GenBank #FJ824669). Thus, these primers were located within conserved sequence stretches flanking the regions of interest but include nucleotide sequences characteristic of *P. malariae*/*P. brasilianum*. Primers nested within the first primer pair were also designed and were used in a nested protocol in order to increase the sensitivity of detection, since *P. malariae* or *P. brasilianum* infections usually present very low parasitemias. All oligonucleotides were checked for specificity by using the Primer-BLAST tool provided by the National Center for Biotechnology Information (http://www.ncbi.nlm.nih.gov/tools/primer-blast/). In experimental PCR, these oligonucleotides were also specific for *P. malariae/P. brasilianum* DNA, showing no amplification with DNAs from other *Plasmodium* species or hosts as templates (data not shown). Table 3 contains the sequences of all oligonucleotides used herein.

**Table 3:** PCR primers and amplification conditions

Region	PCR primers	Amplification conditions	Amplicon size^a^
*Fragment 1*			
First Reaction	F:5′-GCA TCT AAA TGG CTA CTG TGA TAT AC-3′	94 °C 5 min (1×); 94 °C 1 min, 53 °C 2 min, 72 °C 2 min (25×); 72 °C 5 min (1×)	595 bp
	R:5′-GGC ATC TGT AAA TAG ACC ATC C-3′		
Nested Reaction	F:5′-TAA TGA AAA GGA ATT AGA AAT G-3′	94 °C 5 min (1×); 94 °C 1 min, 43 °C 2 min, 72 °C 2 min (30×); 72 °C 5 min (1×)	533 bp
	R:5′-CCA TAT TGA ATA CTA TAT TTT TC-3′		
*Fragment 2*			
First Reaction	F: 5′-CCA TAC TAT TTA ATT GCA CTA AAG-3′	94 °C 5 min (1×); 94 °C 1 min, 58 °C 2 min, 72 °C 2 min (25×); 72 °C 5 min (1×)	723 bp
	R:5′-ACA CAC ATA AGC AGT TTT CAA AAA G-3′		
Nested Reaction	F:5′-AGG GAA ATT GAT AAA TTA AAT ATT TC-3′	94 °C 5 min (1×); 94 °C 1 min, 58 °C 2 min, 72 °C 2 min (30×); 72 °C 5 min (1×)	666 bp
	R:5′-CTT TTC AAG ATA TTG CAA TTT GGA-3′		
*Fragment 3*			
First Reaction	F:5′-GCA ATG TTC TAC AAA ATC AGT ACA AAG-3′	94 °C 5 min (1×); 94 °C 1 min, 53 °C 2 min, 72 °C 2 min (25×); 72 °C 5 min (1×)	765 bp
	R:5′-CTA CAA AAG CTG CTA GTA CAT GTC T-3′		
Nested Reaction	F:5′-GAA GAA GAA TGA CAA ACT TAA AAA C-3′	94 °C 5 min (1×); 94 °C 1 min, 43 °C 2 min, 72 °C 2 min (30×); 72 °C 5 min (1×)	632 bp
	R:5′-CTT TTA AAA TAC TAT ATT CTT TAA TAT G-3′		
*Fragment 4*			
First Reaction	F:5′-GAG GTG ATC GTG TTT CCC ATT G-3′	94 °C 5 min (1×); 94 °C 1 min, 48 °C 2 min, 72 °C 2 min (25×); 72 °C 5 min (1×)	532 bp
	R:5′-CAT TTT CAG TTT TTT TAT CAC CC-3′		
Nested Reaction	F:5′-GTA AGA AAG AAA AGG AAA ATC CAT TAG-3′	94 °C 5 min (1×); 94 °C 1 min, 49 °C 2 min, 72 °C 2 min (30×); 72 °C 5 min (1×)	443 bp
	R:5′-CTG AGT CTT GTA CAA CTT GGT C-3′		
*Fragment 5*			
First Reaction	F:5′-GAC CAA GTT GTA CAA GAC TCA G-3′	94 °C 5 min (1×); 94 °C 1 min, 48 °C 2 min, 72 °C 2 min (25×); 72 °C 5 min (1×)	737 bp
	R:5′-GTA AGT TAA ACA TAA TTA ATA AAG CTG-3′		
Nested Reaction	F: 5′-GGG TGA TAA AAA AAC TGA AAA TG-3′	94 °C 5 min (1×); 94 °C 1 min, 49 °C 2 min, 72 °C 2 min (30×); 72 °C 5 min (1×)	631 bp
	R:5′-GCC GAG GAA ACT TGA AGA AC-3′		

^a^This size is estimated based on that of the sequence from isolate MM1 deposited in GenBank (#FJ824669)

### Amplification and sequencing of the PmMSP1

Primer sequences and amplification conditions used to obtain each fragment and the resulting sizes of the amplicons are shown in Table 3. Each 25 μl reaction mixture for nested1 amplifications contained 5 μl of DNA template, 250 nM of each primer, 2 mM MgCl_2_, PCR buffer (50 mM KCl, 20 mM Tris–HCl, pH 8.8), 0.125 mM each of dATP, dCTP, dGTP, dTTP, and 0.5 units of Platinum® *Taq* DNA Polymerase (Invitrogen). One microliter of the nested1 amplification product served as the DNA template for each of the 25 μl nested2 amplifications. The concentration of the nested2 primers and other constituents were identical to nested1 amplifications. The PCR products of nested2 amplifications were submitted to electrophoresis in 1 % agarose gel stained with 5 × 10^5^-diluted GelRed nucleic acid stain (Biotium, Hayward, CA, USA). PCR fragments were purified and directly sequenced. For fragment 2, due to the presence of the microsatellite, PCR-fragments were cloned in pGEM-T Easy Vector (Promega) and positive clones were sequenced. Sequencing was performed using the Big Dye Terminator v3.0 Cycle Sequencing Kit in an ABI Genetic Analyzer (ABI, USA). The sequences obtained were aligned with the published *P. malariae msp1* sequence (FJ824669) using Clustal X version1.81.

### Phylogenetic analysis

Phylogenetic analysis of *msp1* sequences was performed by maximum likelihood using RAxML v. 8.1.6, with data partitioned in three sets, based on codon position. The GTR model with gamma-distributed substitution heterogeneity rates was used, with base frequencies estimated by maximum likelihood, and 1000 bootstrap pseudoreplicates to assess clade support. The substitution model used was chosen based on a preliminary jModelTest analysis [28], which selected model TPM3uf + I + G; since this model is not available in RAxML, we have used the closest model available (GTR + G), without a proportion of invariants in order to avoid modeling the same aspect of molecular evolution twice. The tree was drawn in Dendroscope [29], with cosmetic adjustments performed in Inkscape (http://www.inkscape.org). The resulting protein sequence alignment and phylogenetic tree are deposited in TreeBase under the accession number S18381.

### Ethical considerations

The study protocol was approved by the Research Ethics Committee of the Institute of Biomedical Sciences of the University of São Paulo, Brazil (CEPSH 749/2006 and 065/2008), which included getting patient consent for research use of their blood samples. All the procedures adopted in this work fully complied with specific federal permits issued by the Brazilian Ministry of the Environment (SISBIO, process numbers 18861–3 and 24319–3).

## Results

### Amplification and sequencing of MSP1 fragments from *P. malariae*/*P. brasilianum* isolates

**Fig. 2:**
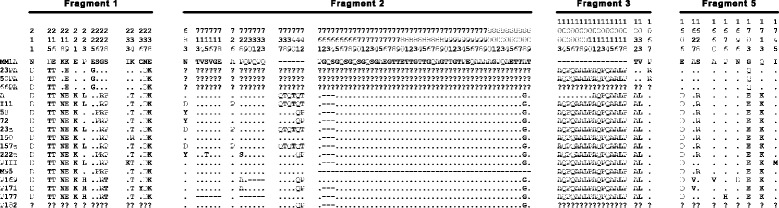
Amino acid polymorphism in merozoite surface protein 1 sequences of *P. malariae* or *P. brasilianum* isolates

Five regions of the *msp1* gene, totalizing ~3000 bp, were amplified and sequenced for the samples presented in Table 1 (GenBank accession numbers JX045639-JX045645 and KR072215-KR072284). In addition to these samples, other *P. malariae* specimens were also tested, but proved negative for PCR amplification of the *msp1* gene. The quantity of target DNA was likely too small in these samples, even though *P. malariae* was positive by nested PCR of the ssrRNA genes. This probably occurred because ssrRNA genes are multiple copy and *msp1* is possibly a single copy gene in *P. malariae*, similar to other *Plasmodium* species. There are also some isolates with sequences of some fragments missing, since genomic DNA from these samples was not sufficient to perform all amplification reactions (Fig. 2).

### Sequence diversity seems limited to specific regions inside analyzed fragments

Fragment 1 encodes a 177 residue peptide in our samples, corresponding to amino acids 160 to 336 in the unaligned MM1 sequence (GenBank #FJ824669). Only the region between amino acids 211 and 238 was polymorphic in our alignment (Fig. 2). Seven different amino acid sequences were found for this fragment. The three samples from the Amazon Region were identical in this *msp1* region and different from all the rest. The samples from *P. brasilianum* were similar to each other and different from human samples but closer to those from Atlantic forest (Fig. 2). Fragment 2 corresponds to amino acids 637 to 858 in the MM1 sequence, but the polymorphic amino acids were found only between positions 683 and 809 of the alignment (Fig. 2). Ten different amino acid sequences were found in this fragment with sizes ranging from 173 to 225 residues. Unfortunately, it was not possible to determine the sequences of the human isolates from the Amazon Region due to the limited amount of genomic DNA available. Many indels were found in relation to the MM1A sequence. There is also a region of a six amino acid indel in four of our sequences from human isolates collected in the Atlantic forest, while six others have only two extra amino acids in comparison to the MM1A sequence. The sequence from mosquito-derived material presented a great deletion in this region (49 amino acids). From the eight *P. malariae* samples, five different sequences were recorded, while each of the five *P. brasilianum* samples presented a unique sequence.

For fragment 3, corresponding to amino acids 950 to 1159 (210 aa) in the MM1 sequence, only the region between the amino acids 1003 and 1067 was polymorphic in our samples (Fig. 2). An insertion of 8 or 16 residues (either one or two copies of AQPQAALP) in relation to the MM1 isolate was found in all of our sequences. However, only three different sequences were found for this fragment. The three samples from the Amazon Region were identical in this *msp1* region and different from all the rest due to just one amino acid (R in position 1067). *P. brasilianum* samples were identical to each other and also to human isolates from the Atlantic forest (excluding the sequence from isolate A, which presented the only 8 amino acid insertion). Fragment 4, with 147 residues corresponding to amino acids 1366 to 1512 in the MM1 sequence, was found to be monomorphic in all the isolates analysed in this study, including *P. malariae* and *P. brasilianum*, from mosquito, simian or human hosts (Fig. 2). Even the nucleotide sequence was the same as that found for isolate MM1, from Cameroon.

In the C-terminal region, fragment 5 was amplified, corresponding to amino acids 1527 to 1736 (210 aa) in the MM1 sequence. Its variability was restricted to 9 amino acids, between residues 1606 and 1745 (Fig. 2). The MSP1_19_ region corresponds to 89 amino acids, between positions 1641 and 1729. Sequences from Amazonian isolates differed from the sequence from Cameroon only by a glutamine residue (position 1703) that was not found in other sequences from Brazil. Sequences from Atlantic forest hosts presented three conserved amino acids: D, E and K, respectively in positions 1606, 1703, and 1713 that were also found in the Amazonian simian isolates from Brazil and Peru. All *P. brasilianum* sequences were unique, and the one from Peru presented a methionine in position 1745 that was the only polymorphism found in this position.

A more quantitative presentation of the amounts of differences between *msp1* fragments analyzed in this work is presented both for each separate fragment as well as in aggregated form representing the concatenation of all fragments (Additional file 1).

### Similar haplotypes in related infections from different patients

Regarding the malaria case acquired by blood transfusion previously reported [21], the *P. malariae* sample obtained from the donor (72) showed the same sequence as the recipient’s (58). This provides additional evidence that the donor and recipient were infected with the same strain, as expected.

### Phylogeny analysis shows genetic and geographic structuring of isolates

**Fig. 3:**
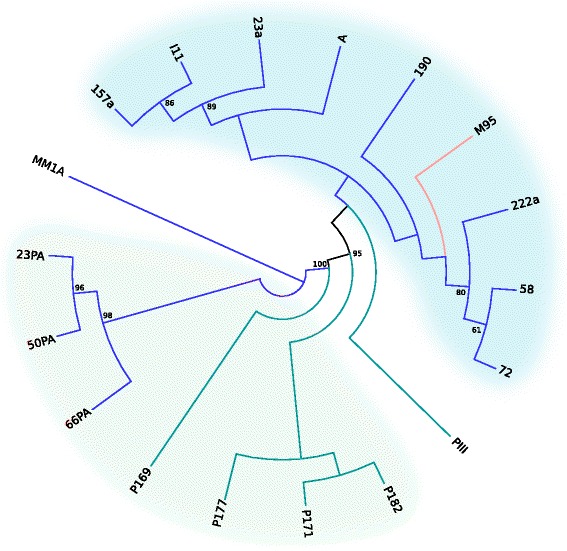
Maximum likelihood phylogeny pattern (branch lengths not displayed) of *msp1* sequences of *P. malariae* (human samples, dark blue branches), *P. brasilianum* (simian samples, light blue branches) and *P. malariae/P. brasilianum* (mosquito sample, red branch) isolates from Cameroon (MM1), Brazilian Amazon Region (green background) or Atlantic forest (blue background). Names of isolates are given according to Table 1. Numbers on nodes indicate bootstrap support values in percentage. Only values above 50 are shown

Figure 3 shows the result from the partitioned maximum likelihood phylogenetic analysis, with branch lengths omitted due to the very high level of sequence similarity between samples (the expected substitution per site bar is 0.001 units long). We found a strong genetic and geographic structure in the phylogeny. The *msp1* sequences were segregated according to their geographic origin and hosts. Regarding sequences obtained from human isolates from the Amazon, we found a well-supported clade that separates them from all other Brazilian sequences. The *msp1* sequences obtained from human isolates collected in the Atlantic forest were also clustered in a clade that was positioned near sequences from simian isolates obtained in the Amazon Region, forming a well-supported clade with a high support value. In general, clades presented low (<50) support values, due to the very high similarity among sequences from the same geographic origin or hosts and the consequent lack in phylogenetic signal (sequences that are too similar do not present enough differences to allow their separation in reliable clades). A Bayesian inference was also performed and showed similar results.

## Discussion

This work presents the first molecular epidemiological data based on the *msp1* gene for *P. malariae/P. brasilianum* samples from Brazil that were obtained from different hosts and from different gene regions. Except for fragment 4, which was monomorphic in our samples, all sequences were different from the MM1A sequence, which was found in a human isolate from Cameroon [17]. As expected, the most polymorphic region was found in fragment 2, already known for including imperfect repeats [17]. Analysis of the percent similarity among the fragment 2 amino acid sequences from all isolates showed identities ranging from 89 to 100 %, and the lowest percentage of identity (89 %) was found between sequences obtained from simian and human samples. Similar results were found in another region of the *msp1* gene (upstream from this work’s fragment 1, corresponding to amino acids 12 to 140 in the MM1 sequence), which showed a polymorphism limited with identities ranging from 93 to 100 % between *P. malariae* and *P. brasilianum* sequences from French Guiana [6].

The repeats on fragment 2 facilitate recombination events, making this region useful as an informative genetic marker. Our results have also shown that, most likely, an absence of polymorphism in this *msp1* region reflects stability also in other regions. The corresponding region in *P. falciparum* and *P. vivax* proved to be likewise the most polymorphic one in many studies [14, 20, 30–32]. However, as opposed to what has been found with *P. falciparum*, our study indicates a likely absence of allelic dimorphism in *P. malariae* and *P. brasilianum msp1* genes, as seen in other *Plasmodium* species [18, 33]. Nevertheless, the possibility of some sequences being so different that our oligonucleotides would be unable to amplify them cannot be ruled out. In this case, the domain architecture would be fundamentally different between *P. malariae/P. brasilianum* and other *Plasmodium* species analyzed so far. It is important to note that, more recently, low levels of sequence diversity at the *msp1* locus have also been found for *P. ovale curtisi* and *P. ovale wallikeri* from Thailand [18].

Blood stages of *Plasmodium* are haploid and the detection of multiple sequences in a human or simian sample indicates the presence of multiple genotypes. However, we also tested a sample from a mosquito, where the *Plasmodium* stages can be haploid (sporozoites) or diploid (ookinete and oocyst). Since multiple sequences were not detected, we could assume that the infections are clonal or there is just one infection in haploid stage. As the mosquitoes were used without severing of head/thorax (sporozoites) from abdomen (ookinete and oocyst), this cannot be determined. These results were obtained with sequencing of cloned fragments and confirmed also by sequencing directly from PCR. Interestingly, the fragment 2 sequence obtained from the mosquito *Plasmodium* sample showed a rather different size, presenting a region with a large deletion (about a third of the amplified sequence), but was highly conserved in its sequence when compared to sequences found in human isolates of the same region, indicating a likely recombination event in this hotspot region.

The main origin of the isolates used in this study was an area with persistently low prevalence of *P. malariae* and *P. vivax* located at the Atlantic forest region of São Paulo State [9]. The low variability of the *msp1* sequences of these isolates indicates the likely “clonal” source and low possibility of future heterologous recombination. In fact, as well as for *msp1* sequences from *P. ovale* [18], the same *P. malariae* haplotype was identified in isolates collected many years apart and in different locations. Considering that the range of flight for *An. (Ker.) cruzii* is roughly 1 km [34], the spread of the same haplotype could be attributable to human movement. The low level of *msp1* sequence diversity from *P. malariae* could indicate, besides the low transmission rate, events of genetic bottlenecks probably caused by malaria control activities.

### Relationship of *msp1* sequence with geographical features and hosts aiming to give insights into the evolutionary and phylogenetic relationships of *P. brasilianum* and *P. malariae*

In terms of geographical origin, *msp1* sequences were informative to distinguish the two main sites of sample origin (Amazon region and Atlantic forest), grouping them in two different clades. Similarly, in relation to the hosts, sequences from parasites with the same host species were clustered. According to the phylogenetic analysis, the sequence obtained from the mosquito sample was included in the group of human isolates from the Atlantic forest, indicating its probable source of infection. Interestingly, sequences from human parasites isolated from the Atlantic forest clustered with the sequences from simian parasites isolated from the Amazon region instead of with those from human parasites isolated in the Amazon region. This indicates that *P. malariae* from human residents in the Amazon Region are genetically different. Their clustering in the phylogenetic tree, with the human isolate from Africa could indicate its origin and also would explain why many of these infections are symptomatic [35]. On the other hand, for the Atlantic forest region, the clustering of sequences from human isolates with sequences from simian isolates supports the idea of zoonotic malaria in this region [7], although it is very likely that it is being currently maintained in the region due to asymptomatic individuals who are acting as main source of parasites [36].

The high genetic identity found between *P. malariae* from humans and *P. brasilianum* from New World primates suggests the occurrence of a recent transfer between these hosts [3–5], but there is no evidence to determine whether the transference occurred from monkeys to humans or vice versa. The direction of the host transfer can then be suggested by comparing the genetic diversity of the human and primate parasites [37]. Although studies focusing on these species are rare, some works indicated that *P. brasilianum* strains seem to show greater divergence than *P. malariae*, thus *P. malariae* could derive from *P. brasilianum* [37, 38]. In this work, we also identified greater diversity among *P. brasilianum* isolates that could support this hypothesis. In contrast, *msp1* sequence analysis of *Plasmodium knowlesi* from humans and macaques in Thailand revealed that the diversity of human-derived sequences exceeded that of monkey-derived sequences [39].

On the other hand, in the case of *P. malariae*, the host switch between humans and monkeys might have occurred through a single transfer associated with a bottleneck, after the European colonization of the Americas in the 16th century [3] and thus, *P. brasilianum* would be derived from *P. malariae*. An analysis of the sequence sizes in fragment 2 could support this hypothesis. It has been thought that repeats present in longer stretches are likely to be older than short ones, as the former needs more time for accumulation of base substitutions [40]. From this point of view, our data suggests that the simian sequences (with shorter stretches in this work) could have derived from the human sequences (longer stretches in this work). Anyway, microsatellite evolution is a dynamic process during which repeats may shrink as well as expand over an evolutionary time scale [41].

Recently, *P. malariae* has been found from chimpanzees in West [42] and Central Africa [43, 44] and bonobos in Central Africa [45]. However, the mitochondrial genomes of the parasites related to *P. malariae* found in bonobos carry a six nucleotide insert that has not been observed for *P. malariae* or the South American parasite *P. brasilianum* [45]. Analysis of *msp1* genetic polymorphism in such isolates could potentially shed light on the question of origin and host switch for these parasites.

The assumption that infection diversity and multiplicity is positively correlated with the frequency of transmission is reasonable [46]. Low allelic diversity was detected in Brazilian isolates, which seems to reflect a very low transmission rate. It has been established that *P. malariae* shows a reduced growth rate [47] and one hypothesis proposed that the low parasitemia obtained *in vivo* may be caused by its preference for mature erythrocytes [48]. *P. malariae* is also known to promote infections that are frequently long-lasting (up to 40 years absence from reinfection) and non-symptomatic [49]. Exclusive infections with *P. malariae*, like those found in our study area, would also explain the very low transmission rates seen in this region. In other locations, where *P. malariae* occurs in coinfections, mainly with *P. falciparum*, it has developed increased transmission efficiency in the presence of a coinfecting parasite of another species [50]. In areas of high transmission of all species, *P. falciparum* is dominant for a while and is then being succeeded by *P. vivax*, which holds center stage for some time, then being replaced by *P. malariae*, causing a “residual infestation” [51]. At the end of the XIX century, malaria (caused by *P. falciparum*, *P. vivax* and *P. malariae*) was present throughout the entire Brazilian territory, but after a successful World Health Organization (WHO) malaria eradication campaign, initiated in 1956, the number of cases decreased drastically outside the Brazilian Amazon. Currently, malaria transmission is primarily concentrated in the Amazon. A residual, low malaria transmission (0.05 % of all Brazilian malaria cases), occurs in the Atlantic Forest, caused by *P. vivax* or *P. malariae* [9]. Thus, *P. malariae* uses its long association with man ‘to learn’ and to cope with difficulty and succeed [51].

## Conclusions

Evidence of geographical clustering of haplotypes was verified even though using a small sample available. However, further analysis with other Brazilian samples of different geographical origins is important to characterize the epidemiology of Brazilian *P. malariae* and *P. brasilianum* isolates in a higher resolution. Comparisons between human and simian *Plasmodium* isolates are also of interest, particularly in areas of the Atlantic forest where humans and monkeys are at risk of malaria transmission by the same anopheline vectors. Additional studies concerning the characterization of antigenic diversity in vaccine candidate antigens are valuable for future vaccine trials as well as for understanding the population dynamics of *P. malariae* and *P. brasilianum* parasites.

## Additional file

Additional file 1:
**Pairwise similarity matrix among** ***P. malariae/P. brasilianum*** **isolates based on the MSP1 protein sequence fragments**. The matrices on the left show, for each fragment separately, the raw numbers of identical amino acids and total amino acids compared between each pair of isolates. The matrices on the right show the aggregated similarity or difference values for the concatenated regions.(XLS 40 kb)
